# Cost-minimization analysis of subcutaneous versus intravenous trastuzumab administration in Chilean patients with HER2-positive early breast cancer

**DOI:** 10.1371/journal.pone.0227961

**Published:** 2020-02-05

**Authors:** Luis Rojas, Sabrina Muñiz, Lidia Medina, Jose Peña, Francisco Acevedo, Mauricio P. Pinto, Cesar Sanchez

**Affiliations:** 1 Departamento de Medicina Interna, Facultad de Medicina, Pontificia Universidad Catolica de Chile, Santiago, Chile; 2 Unidad Docente de Farmacología y Toxicología, Facultad de Medicina, Pontificia Universidad Catolica de Chile, Santiago, Chile; 3 Departamento de Oncologia, Complejo Asistencial Dr. Sotero del Rio, Santiago, Chile; 4 Centro de Cancer “Nuestra Señora de la Esperanza”, Red de Salud UC CHRISTUS, Pontificia Universidad Catolica de Chile, Santiago, Chile; 5 Departamento de Hematologia y Oncologia, Facultad de Medicina, Pontificia Universidad Catolica de Chile, Santiago, Chile; Harran Universitesi, UNITED STATES

## Abstract

**Purpose:**

Trastuzumab (TZM) improves survival and the risk of recurrence among patients with early-stage HER2+ breast cancer (BC). TZM treatment can be given intravenously (IV-TZM) or subcutaneously (SC-TZM). Although both methods have similar efficacy and safety, they differ in dosage and administration. Previous studies of cost minimization determined that SC-TZM is associated with lower costs than IV-TZM; however, those studies did not include the costs associated with body weight-based dosage and the treatment of adverse drug reactions (ADRs).

**Methods/Patients:**

We performed a model-based cost-minimization analysis. The analysis included direct and indirect medical costs associated with TZM preparation (adjusted by body weight) and administration and also costs due to severe ADRs and non-medical costs that occurred during the total treatment course (18 cycles). We performed a sensitivity analysis to test the robustness of the results across various TZM costs and patient body weights.

**Results:**

The overall cost (in USD) of IV-TZM treatment was $83,309.1 per patient compared with $77,067.7 per patient for SC-TZM. Thus, one year of SC-TZM treatment cost $6,241.4 less per patient than one year of IV-TZM treatment. The sensitivity analysis revealed that the results were mainly driven by the price of each TZM vial and body weight.

**Conclusion:**

SC-TZM is a cost-saving therapy for Chilean patients with early-stage HER2+ BC. Given their similar efficacy and safety, we suggest the use of SC formulations rather than IV formulations. The use of SC-TZM instead of IV-TZM may have a significant economic impact on public/private healthcare systems.

## Introduction

Worldwide, breast cancer (BC) is one of the most prevalent malignancies among women. More than one million new BC cases are diagnosed every year, affecting 43.3/100,000 women and causing >500,000 deaths with a mortality rate of 12.9/100,000 women [1]. In Chilean women, BC is the leading cause of cancer death. Despite great advances in treatment, more than 1,300 women died in Chile from BC in 2012, with an estimated mortality rate of 15.69/100,000 women [2,3]. The World Health Organization classifies BC as a public health problem at a global scale. About 15–25% of BC cases display amplification and/or overexpression of Epidermal Growth Factor Receptor Type-2 (HER2) [3,4], an oncoprotein that interacts with cell-proliferation pathways to grant protection against apoptosis. HER2 positivity is commonly associated with increased aggressiveness and poor prognosis [3–5].

Trastuzumab (TZM) is a recombinant monoclonal antibody that binds to the outer domain of HER2 with high selectivity and affinity [4]. The mechanism of TZM action involves inhibition of the HER2 intracellular domain, which interferes with cancer cell growth by preventing the activation of cell-proliferation or apoptosis signals [5,6]. The use of TZM in combination with chemotherapy (CT) in the treatment of early-stage HER2+ BC improves survival by up to 35% (with 1 year of TZM use) and reduces the risk of recurrence by 33–52%, including loco-regional recurrence [1,4,5,7,8]. Hence, TZM is the current standard treatment for early and advanced HER2+ BC [9]. Previously, the only administration route for TZM was intravenous infusion (IV-TZM), which requires an initial administration of an 8 mg/kg loading dose over 90 min, followed by 17 cycles of a 6 mg/kg maintenance dose administered over 30–90 min every 3 weeks for 1 year [4–6]. That scheme requires healthcare professionals to spend a substantial amount of time on drug preparation and administration procedures. IV-TZM treatment is also a time-consuming procedure from the patients’ perspective [1]. The United States food and drug administration (FDA) approved the use of subcutaneous TZM (SC-TZM) in 2013. The subcutaneous formulation is administered in a single fixed dose of 600 mg over a relatively short time and does not require a loading dose or dose adjustments according to the patient’s body weight, meaning a much simpler preparation and more stable doses during the course of treatment, which still involves 18 doses administered over 1 year [1,6]. Thus, compared with IV-TZM, SC-TZM is associated with better optimization of time and financial resources for both patients and medical staff [4,5].

Previously, clinical trials have demonstrated that SC-TZM and IV-TZM display similar pharmacokinetics, efficacy and safety profiles [10,11],. The multinational *enHANced treatment with NeoAdjuvant Herceptin (HannaH)* study compared the efficacy and safety of SC-TZM with those of IV-TZM (both in combination with CT) and reported similar pharmacokinetic parameters, complete response rates, response times, and adverse drug reactions (ADRs) between the two treatment modalities. The study concluded that SC-TZM is an effective alternative to IV-TZM and, given its simplicity and ease of use, might represent a cost-effective and time-saving choice for patients and medical staff [12,13].

The cost of pharmaceutical formulations is a key factor when selecting and implementing a therapeutic intervention in any population, especially when there are alternatives with similar therapeutic efficacy [14]. Cost-minimization analysis is a useful tool in such situations [15]. Previous cost-minimization studies demonstrated that SC-TZM is associated with lower medical and non-medical costs than IV-TZM. Those studies did not include ADR-associated costs, however. Additionally, the costs were not adjusted for patients’ body weight, which is a key variable that determines the number of drug vials required and, ultimately, the final cost of the therapeutic regime. Remarkably, no cost-minimization studies of SC-TZM and IV-TZM have been performed in Latin America, making it difficult to extrapolate the findings of previous studies to countries in that region. The goal of our study was to compare the direct and indirect medical and non-medical costs associated with SC-TZM and IV-TZM in patients with HER2+ early BC in a private health center in Chile.

## Materials and methods

### Ethics approval

The Scientific & Ethics committee at the Pontificia Universidad Catolica de Chile approved this study (approval number #151205001, dated on December 10th, 2015). Given the nature of our work no sensitive information was collected from patients and all data were anonymized.

### Cost-minimization analysis

This study was conducted in a single private hospital. Assuming equivalent clinical efficacy between SC-TZM and IV-TZM [12], we developed a cost-minimization analysis using a model that incorporates the non-medical costs and the direct and indirect medical costs of 1 year of therapy with SC-TZM or IV-TZM in patients with HER2+ early BC. All treatments were administered according to the treatment protocols of Nuestra Señora de la Esperanza Cancer Center, which is part of the Red de Salud UC-Christus network. The treatment protocol for IV-TZM was an initial dose of 8 mg/kg body weight followed by 17 maintenance doses of 6 mg/kg body weight with an interval of 3 weeks between doses. The protocol for SC-TZM treatment was 18 fixed doses of 600 mg with an interval of 3 weeks between doses. We did not include the costs related to adjuvant CT and central venous access devices central venous access devices in the analysis. The costs of TZM in our analysis were based on the price of Herceptin (Roche), because biosimilar forms of the drug are not yet available in Chile.

### Time horizons

This study was based on a single year of therapy, meaning a total of 18 TZM cycles/doses. The costs were based on 2017 economic variables and expressed in United State Dollars (USD). The economic evaluation in this study covers a 1-year time horizon, so no discounting procedure was performed.

### Cost estimates

We created a spreadsheet model to assess the costs of SC-TZM and IV-TZM. The costs included direct and indirect medical costs associated with the work time of healthcare professionals, drugs, and consumables used to prepare and administer IV-TZM or SC-TZM; treatment costs for severe ADRs; and non-medical costs associated with lost work time and time spent by patients on transportation between the workplace and the hospital. The model assumed that all patients underwent the same preparation and administration protocols. Costs were calculated on a per-patient basis and also for 100 patients, because that is close to the number of patients treated with TZM at Red Salud UC Christus over the last 3 years. The unit costs used in the pharmacoeconomic analysis are shown in **Table 1**.

**Table 1:** Associated costs for preparation and administration of a single dose (costs in USD 2017).

	Resource	USD
**Preparation**	Saline 250 ml (IV only)	$2.4
	IV tubing (IV only)	$5.3
	Needle	$0.4
	Disposable syringe 5 ml IV (IV only)	$0.5
	Disposable syringe 5 ml SC (SC only)	$0.2
	Red cap sterile luer	$0.1
	Pharmacist time and infrastructure	$35.6
	Trastuzumab vial IV (IV only)	$3,606
	Trastuzumab vial SC (SC only)	$4,228
**Administration**	Infusion pump set (IV only)	$20.7
	Syringe 5mL Heparin (IV only)	$3.1
	Disposable sterile gloves (IV only)	$1.5
	Swab stick	$0.8
	Port needle (IV only)	$38.6
	Gauzes (IV only)	$0.2
	Saline 250 mL (IV only)	$2.4
	Nurse time (1 h)	$10.2
	Chair time (0–1 h)	$98.7
	Chair time (2–3 h)	$172.7

SC: subcutaneous; IV: intravenous

#### Preparation costs

The preparation costs included the costs of drugs, equipment, and consumables, and pharmacist time. Both IV-TZM and SC-TZM were given over 18 treatment cycles. Because the IV-TZM loading and maintenance doses are dependent on the patents’ body weight, we used anonymous body weight data collected for the patients in our cancer center to calculate the number of TZM vials used for each patient in each IV-TZM treatment cycle. In Red Salud UC-Christus, the remaining contents of IV-TZM vials after a single treatment cycle cannot be used for subsequent treatment cycles or for other patients. No adjustment was made in the analysis for the potential waste of materials associated with the discarding of remnant portions of drug from partially used vials. The costs of all consumable materials used for IV-TZM reconstitution and other drug preparation and storage activities (including the equipment and facilities used) were estimated. The cost of pharmacist time was estimated by multiplying the time spent on drug reconstitution, preparation, and storage by the pharmacists’ wage. In order to calculate pharmacist’s cost, the number of work hours was multiplied by the estimated average salary of the Red Salud UC- Christus hospital’s pharmacists.

#### Administration costs

The administration costs included consumables used to administer treatment, infusion chair time, nurse time required to administer treatment, and non-medical costs incurred by patients while attending treatment. The consumables included saline solution, IV tubing, port-needles, syringes, sterile gloves, scalp vein tips, red cap sterile luers, gauzes, swab sticks to clean the skin, and infusion pump sets. Almost all of the patients had central venous access devices for infusion chemotherapy. We assumed in the analysis that 100% of the patients treated with IV-TZM had such a device. The infusion chair time was the time from when the patients first sat in down in the infusion chair to the time they rose from the chair at the end of each treatment cycle. For IV-TZM, the infusion chair time was about 160 min for the first three cycles (90 min for infusion and 60 min to monitor potential ADRs) and 60 min for each subsequent cycle (30 min for infusion and 30 min to monitor potential ADRs). For SC-TZM, the time required for infusion and surveillance was about 35 min for the first three cycles and 15 min for each subsequent cycle. Nurses’ time costs associated to administration of treatment was estimated by multiplying average salaries for Nuestra Señora de la Esperanza Cancer Center nurses. Non-medical costs for patients included costs related to transient work absenteeism, which were estimated from the amount discounted from wages for the time spent on transportation to and from the hospital (estimated to be 100 min per patient per treatment cycle), the time spent in the treatment room, and the total time spent in the hospital. Work productivity losses were estimated on the basis of the employment rate and average salary among Chilean women in the age range that usually receives TZM treatment. The occupational and wage data were obtained from the National Institute of Statistics on the Metropolitan district population [16]. Our analysis did not include the costs associated with an accompanying partner, because the therapies that the study participants received do not usually affect patient autonomy.

#### Treatment costs for ADRs

Treatment costs due to severe ADRs associated with IV-TZM or SC-TZM were estimated using previously quantified ADR rates ([12] and **Table 2**). Although left ventricular dysfunction is not considered a serious ADR, we included it in our analysis because it requires considerable economic interventions: at least two medical evaluations, two echocardiograms, and new loading doses upon restart of therapy after recovery of cardiac function [17]. The treatment protocols included the performance of an echocardiogram every 3 months during the treatment period (part of the protocol for early detection of ventricular dysfunction), the costs of which were also included.

**Table 2:** Incidence of trastuzumab-associated serious adverse reactions.

Adverse Drug Reaction	IV	SC
Fever Neutropenia	3.4%	4.4%
Neutropenia	3%	2.4%
Bronchopneumonia	0.13%	0.13%
Pneumonia	0.13%	0.13%
Tonsillitis	0%	1%
Cellulitis	0%	0.13%
Pleural effusion	0%	0.13%
Lung embolism	0%	0.13%
Dysfuntion left ventricular	3.4% (reversable 3%)	3.7% (reversable 2.7%)

SC: subcutaneous; IV: intravenous

### Health resources

We interviewed the healthcare personnel involved in the preparation and administration of the TZM treatments and also those involved in the management of patients undergoing the treatments in order to understand all of the processes, drugs, and consumables used and the time involved. The healthcare professionals interviewed included oncologists, nurses, pharmacists, and administrative personnel from the Pharmacy Unit and the Nuestra Señora de la Esperanza Cancer Center of the Red de Salud UC-Christus network.

### Sensitivity analysis

We performed a one-way sensitivity analysis to confirm the robustness of our results. The sensitivity analysis included the variables with the greatest impact on the results: the difference in costs between SC-TZM and IV-TZM and the changes in body weight of the patients during therapy (because the dosing of IV-TZM is dependent on body weight).

Because of the nature of our study, an ethics evaluation waiver was granted by the School of Medicine Research Committee of the Pontificia Universidad Católica de Chile. The spreadsheet model and sensitivity analysis were performed using Microsoft Excel 2011 for Mac Version 14.5.2.

## Results

### Costs estimates

#### Preparation costs

The preparation of a single dose of IV-TZM cost $587.3 less per patient than the preparation of a single dose of SC-TZM (**Table 3**). The major contributor to the difference cost was the drug costs; however, the drug costs for IV-TZM depended on the body weight of the patients. Therefore, we performed a weight-adjusted analysis using patient data from the Red Salud UC-Christus health center. The weight data showed that 74% of the patients (those weighing >55 kg) required two loading vials for their initial IV-TZM treatment. In addition, 22% of the patients (those weighing >73 kg) required two maintenance vials for each maintenance IV-TZM treatment. Based on that, the estimated preparation costs for 18 doses of IV-TZM for 100 patients exceeded the preparation costs for 18 doses of SC-TZM for 100 patients by $485,157.87 (**Table 4**).

**Table 3:** Associated costs for preparation of a single dose (costs in USD 2017).

	IV	SC
Trastuzumab vial	$3,438,71	$4,032.41
Consumables	$9.07	$1,7
Pharmacist time	$33,97	$33,97
**Total**	$3,481.75	$4,069.08

SC: subcutaneous; IV: intravenous

**Table 4:** Overall estimated preparation cost for 1 year (18 doses) of IV-TZM or SC-TZM treatment for 100 patients, adjusted for body weight (costs in USD 2017).

	IV dose 1^st^ dose	IV doses 2–18^th^ doses	SC doses 1^st^–18^th^ doses	Difference
Trastuzumab vial	$598,335.8	$7,131,888.2	$7,258,335.88	$471,829,.05
Consumables	$907,4	$15,425	$3,062.65	$13,269.75
Pharmacist time	$3,329,12	$57,750	$61,147.06	$0
**Total**	$7,807,635..4		$7,322,545,6	$485,089..87

SC: subcutaneous; IV: intravenous

#### Administration costs

The total administration costs to treat 100 patients with full courses of IV-TZM and SC-TZM were $348,546 and $200,536 respectively. The difference was mainly derived from the costs of consumables, chair time, and the work time of the healthcare professionals (**Table 5**).

**Table 5:** Overall estimated administration cost for 1 year (18 doses) of IV-TZM or SC-TZM for 100 patients, adjusted for body weight (costs in USD 2017).

	IV	SC	Difference
Consumables	$121,356	$ 1,456	$119,900
Chair time cost	$202,470	$180,508	$21,930
Medical staff active time	$24,720	$18,540	$6,180
**Total**	$348,546	$200,536	$148,010

SC: subcutaneous; IV: intravenous

#### ADR treatment costs

The average costs of ADRs associated with IV-TZM treatment and SC-TZM treatment were $55,725 and $69,148, respectively. The analysis included the minimum and maximum values of the cost due to ADRs (**Table 6**). Despite the substantial costs associated with each ADR, serious ADRs associated with IV-TZM and SC-TZM are rare and consequently have a low relative impact on overall costs compared with the impacts of other evaluated items.

**Table 6:** Average treatment cost for ADRs associated with IV-TZM or SC-TZM treatment (costs in USD 2017).

Adverse effect	Average Cost	
	IV	SC
Fever Neutropenia	$12,680	$14,907
Neutropenia	$11,188	$8,944
Bronchopneumonía	$6,748	$6,744
Pneumonia	$6,748	$6,744
Tonsilitis	—	$2,550
Cellulitis	—	$3,053
Pleural effusion	—	$5,342
Lung embolim	—	$6,833
Dysfunction left ventricular	$18,360	$14,683
Echocardiograms (4)	$101,698.7	$101,698,7
Total	$157,425.	$171,537.
**Difference IV—SC**	$-14,113	

SC: subcutaneous; IV: intravenous

#### Non-medical costs

Our estimates indicate that patients lost on average 40.7 and 58.5 work hours when receiving SC-TZM and IV-TZM treatments, respectively. Based on the employment rate (62%) and average wage of Chilean women in the age range that commonly receives TZM therapies (hourly salary of USD $4.7,) we estimated that the average total productivity loss for 100 women to receive TZM treatment were $ and $47,090 for IV-TZM and SC-TZM, respectively.

#### Total costs

The total costs to treat 100 patients with HER2+ early BC were $8,330,911,1 per year for IV-TZM and $7,706,775.8 per year for SC-TZM. Those results indicate that it cost $624,135,3 less to use SC-TZM to treat 100 patients than to use IV-TZM to treat the same number of patients (**Table 7**). The difference was due to drug preparation costs and the costs associated with the use of consumables and facilities.

**Table 7:** Total estimated costs for 1 year of TZM treatment (18 doses) for 100 patients (costs in USD 2017).

Costs	IV	SC	Difference
Preparation	$7,807,635	$7,322,545.6	$485,089.8
Administration	$348,546	$200,536	$148,010
ADR (average)	$157,424.9	$171,537.5	$-14,112,6
Non-medical costs	$17,340.8	$12,156.7	$5,148.1
**Total**	$8,330,911.1	$7,706,775.8	$624,135.3

SC: subcutaneous; IV: intravenous

### Sensitivity analysis

**Fig 1:**
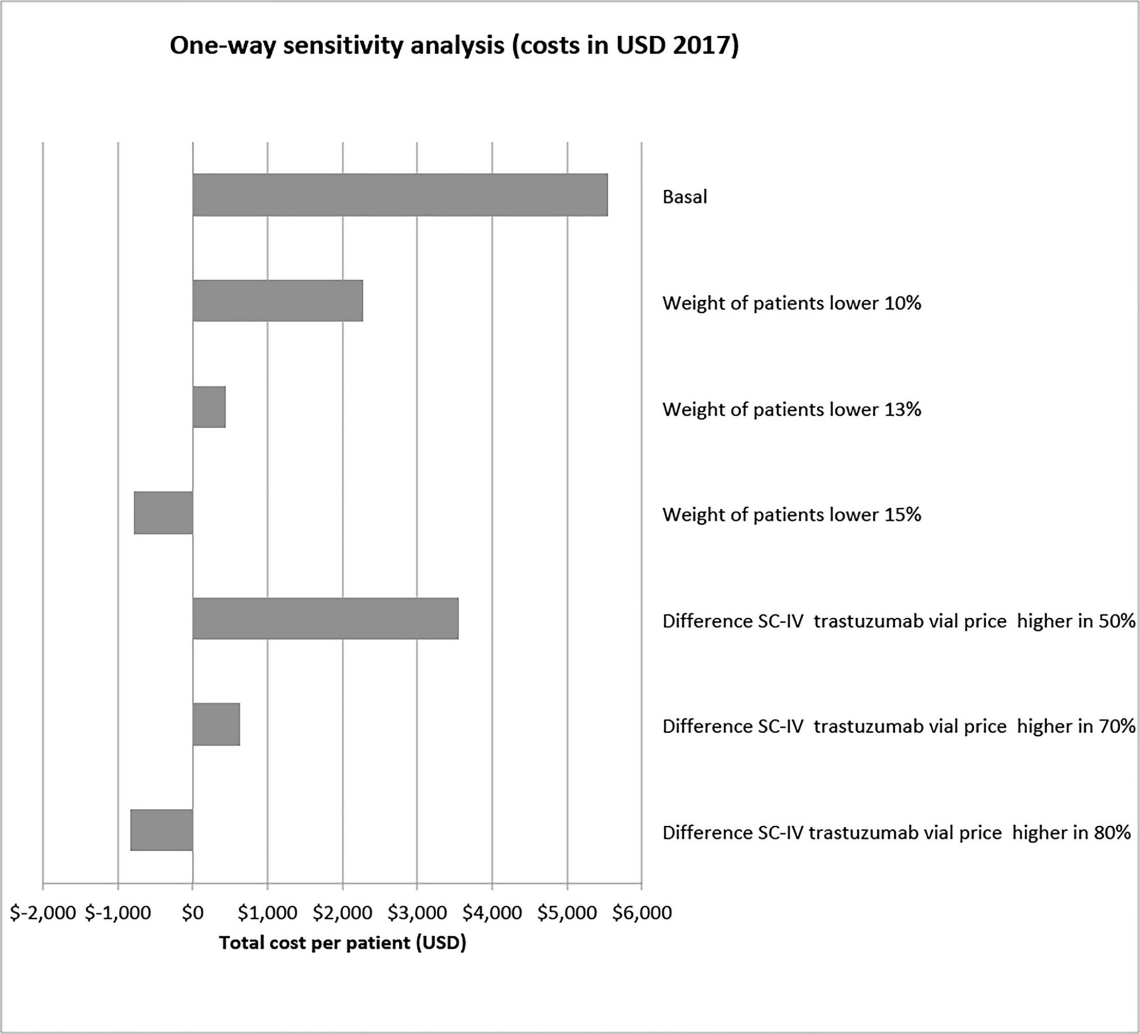
Sensitivity analysis by ADR costs (costs in USD 2017). Abbreviations: Min: minimum; Max: maximum; SC: subcutaneous; IV: intravenous.

The one-way sensitivity analysis showed that IV-TZM treatment would become more economical than SC-TZM if the difference in cost between the drug vials used for IV-TZM and those used for SC-TZM were increased by 80%, or if the average body weight of the patients were reduced by 15% or more during the course of therapy (**Fig 1**).

## Discussion

We compared the estimated cost of 18 cycles of IV-TZM treatment with that of 18 cycles of SC-TZM treatment for patients with HER2+ early BC in a private Chilean health institution. Our analysis included non-medical costs, direct and indirect medical costs associated with drug preparation and administration, and the costs associated with serious ADRs. We found that for the treatment of 100 patients, the use of SC-TZM resulted in a total cost reduction of $624,135.3 ($6,241 per patient) compared with the use of IV-TZM.

The difference in cost between IV-TZM and SC-TZM was mainly controlled by body weight and drug costs. The number of drug vials required for a single round of IV-TZM treatment depends on the patient’s body weight, which can change during the course of treatment. If the patients in our study lost 15% of their body weight on average during the course of treatment, IV-TZM would be the most economical treatment modality. That scenario might be unlikely, because a previous study showed that patients with BC tend to increase their body weight slightly during treatment [18]. Additionally, the use of IV-TZM would result in a cost savings compared with the use of SC-TZM if the difference in the cost of the medications increased by 80%, which might happen if biosimilars become available in Chile and the price of Herceptin does not decrease significantly as a result.

Previous studies reported similar findings. Burcombe *et al*. found that SC-TZM was associated with lower costs than IV-TZM, but their study was an observational study concomitant to the PrefHer clinical trial in which the treatments were not representative of the conditions regularly seen in clinical practice [19]. Lopez-Vivanco *et al*. reported that SC-TZM provided a € 979.60 (USD $1,156) savings per patient versus IV-TZM; however, they did not assess costs associated with serious ADRs, and the criteria used to define patient weights for the calculation of load and maintenance doses were not reported [20]. A cost study performed in Latin America by Romero *et al*. reported favorable figures for SC-TZM, but that study had the same shortcomings as the study by Lopez-Vivanco *et al*. [21]. A review by Papadmitriou *et al*. suggested a substantial economic benefit associated with SC-TZM versus IV-TZM; however, the evidence was somewhat limited (based on only two studies), and the resources employed across the different healthcare systems assessed were highly heterogeneous [22]. Studies by De Cock *et al*. and Lieutnenant *et al*. determined that SC-TZM was associated with lower costs than IV-TZM, but the cost estimates in those studies only included the time patients spent in the treatment chairs and the active time spent by the healthcare personnel to provide the treatment [23,24].

A potential benefit of SC-TZM versus IV-TZM is the increased availability of healthcare facilities and staff, which could be used for other therapies or medical procedures. In the Red de Salud UC-Christus network, TZM is administered to four patients daily on average. If only the SC formulation were used, an additional 4 h of chair time would be available to administer other therapies, which would potentially generate additional earnings of $125.8 per day, or $143,956 per year.

Our analysis has certain limitations. First, the analysis is based on a model that assumes all patients underwent the same processes, however, there could be differences between protocolized processes and real clinical practice Second, all of the data used in our study were obtained from a single institution and therefore can only be extrapolated to other situations with caution. The care processes and costs at Red Salud UC Christus are representative of most private health centers in Chile, so although the absolute costs are not representative of public centers, the qualitative conclusions of our study can be extrapolated to public health centers that have a similar system of patient care.

Our study is one of the first assessments of TZM costs that accounts for serious ADRs that require hospitalization or transient interruption of TZM therapy. Furthermore, our study is one of a few that incorporate patients’ weight to estimate the number of IV-TZM vial and preparation costs for loading and maintenance doses. Finally, this is the first report that includes clinical and economic data from a Chilean hospital.

## Conclusion

Our results suggest that the direct and indirect medical and non-medical costs associated with the preparation and administration of SC-TZM are lower than those associated with the administration of IV-TZM in Chile. The widespread implementation of SC-TZM as an alternative to IV-TZM would potentially impact the budgets of public and private healthcare systems and also have indirect impacts on patients. Given the equivalence in efficacy between SC-TZM and IV-TZM, we encourage the use of SC-TZM instead of IV-TZM for the treatment of patients with HER2+ early BC.

## Supporting information

S1 File Supplementary tables and complete datasets. (ZIP)
