# Supplementary material for: Cost-minimization analysis of subcutaneous versus intravenous trastuzumab administration in Chilean patients with HER2-positive early breast cancer
Source: PLoS One. 2020 Feb 5;15(2):e0227961. doi: 10.1371/journal.pone.0227961 (PMC7001963; doi:10.1371/journal.pone.0227961)
Supplement: S1 File — (ZIP) [file pone.0227961.s001.zip › S1 File/S5 Table.docx]

S5 Table. Estimation of work time absenteeism from the time spent on transportation to and from the hospital, the time spent in the treatment room, and the total time spent in the hospital

| **IV TZM** | First three cycles | Subsequent cycles |
| --- | --- | --- |
| Chair Time per each cycle (hours) | 2,5 | 1 |
| Transportation time per each cycle (hours) | 2 | 2 |
| Total time per each cycle (hours) | 4,5 | 3 |
| Total time 1 patient, 18 cycles (hours) | 58,5 | |
| **SC TZM** | First cycle | Subsequent cycles |
| Chair Time per each cycle (hours) | 0,5 | 0,25 |
| Transportation time per each cycle (hours) | 2 | 2 |
| Total time per each cycle (hours) | 2,5 | 2,25 |
| Total time 1 patient, 18 cycles (hours) | 40,7 | |
