# Supplementary material for: Cost-minimization analysis of subcutaneous versus intravenous trastuzumab administration in Chilean patients with HER2-positive early breast cancer
Source: PLoS One. 2020 Feb 5;15(2):e0227961. doi: 10.1371/journal.pone.0227961 (PMC7001963; doi:10.1371/journal.pone.0227961)
Supplement: S1 File — (ZIP) [file pone.0227961.s001.zip › S1 File/S7 Table.docx]

S7 Table. Non-medical costs obtained from transportation cost and work productivity losses (estimated on the basis to employment rate and average salary among Chilean women in the age range that usually receives TZM treatment)

| Trastuzmab formulation | Cycles | No-medical cost per  1 patient | No-medical cost per  100 patients |
| --- | --- | --- | --- |
| IV | First three cycles | $ 65.6 | $4,064.8 |
|  | Subsequent cycles | $ 213.6 | $13,206 |
| SC | First cycle | $ 14.2 | $880.4 |
|  | Subsequent cycles | $181.9 | $11,276.3 |
