# Supplementary material for: Cost-minimization analysis of subcutaneous versus intravenous trastuzumab administration in Chilean patients with HER2-positive early breast cancer
Source: PLoS One. 2020 Feb 5;15(2):e0227961. doi: 10.1371/journal.pone.0227961 (PMC7001963; doi:10.1371/journal.pone.0227961)
Supplement: S1 File — (ZIP) [file pone.0227961.s001.zip › S1 File/S2 Table.docx]

S2 Table, Estimation of preparation cost of IV-TZM or SC-TZM for 100 patients, adjusted for body weight (costs in USD 2017)

| Trastuzumab forrmulation | Cycles | Number of vials | Number of patients | Costs (USD) |
| --- | --- | --- | --- | --- |
| IV | Loading dose  (1 cycle) | 1 vial | 26 patients | $90,525.65 |
|  |  | 2 vials | 74 patients | $512,114.61 |
|  | Maintenance dosis  (17 cycles) | 1 vial | 78 patients | $4,616,808.30 |
|  |  | 2 vials | 22 patients | $2,588,254.90 |
|  |  |  |  |  |
| SC | 18 cycles | 1 vial | 100 patients | $7,322,545.59 |
