# Supplementary material for: Cost-minimization analysis of subcutaneous versus intravenous trastuzumab administration in Chilean patients with HER2-positive early breast cancer
Source: PLoS One. 2020 Feb 5;15(2):e0227961. doi: 10.1371/journal.pone.0227961 (PMC7001963; doi:10.1371/journal.pone.0227961)
Supplement: S1 File — (ZIP) [file pone.0227961.s001.zip › S1 File/S1 Table.docx]

S1 Table. Estimation of number of patients that required one or more IV trastuzumab vials according to body weight

| Weight | Loading dose | Percentage of patients (%) |
| --- | --- | --- |
| ≤ 55kg | One vial | 26 |
| >55kg | Two vials | 74 |
|  | Three vials | 0 |
|  |  |  |
| Weight | Maintennace dose | Percentage of patients (%) |
| ≤ 73kg | One vial | 78 |
| > 73 kg | Two vials | 22 |
|  | Three vials | 0 |
