# Supplementary material for: Cost-minimization analysis of subcutaneous versus intravenous trastuzumab administration in Chilean patients with HER2-positive early breast cancer
Source: PLoS One. 2020 Feb 5;15(2):e0227961. doi: 10.1371/journal.pone.0227961 (PMC7001963; doi:10.1371/journal.pone.0227961)
Supplement: S1 File — (ZIP) [file pone.0227961.s001.zip › S1 File/S4 Table.docx]

S4 Table. Estimation of chair time and nurse time costs required to administer SC TZM

|  | First cycle | Subsequent cycles |
| --- | --- | --- |
| **Chair time** |  |  |
| Time per each cycle (hours) | 0,5 | 0,25 |
| Cost per each cycle (USD) | $95.6 | $95.6 |
| Cost per 1 patient (USD) | $95.6 | $1,624.6 |
| Cost per 1 patient, 18 cycles (USD) | $1.720,1 | |
|  |  |  |
| **Nurse time** |  |  |
| Time per each cycle (hours) | 1 | 1 |
| Cost per each cycle (USD) | $9.8 | $9.8 |
| Cost per 1 patients (USD) | $9.8 | $166.7 |
| Cost per 1 patients , 18 cycles (USD) | $176.5 | |
