# Supplementary material for: Cost-minimization analysis of subcutaneous versus intravenous trastuzumab administration in Chilean patients with HER2-positive early breast cancer
Source: PLoS One. 2020 Feb 5;15(2):e0227961. doi: 10.1371/journal.pone.0227961 (PMC7001963; doi:10.1371/journal.pone.0227961)
Supplement: S1 File — (ZIP) [file pone.0227961.s001.zip › S1 File/S3 Table.docx]

S3 Table. Estimation of chair time and nurse time costs required to administer IV TZM

|  | First three cycles | Subsequent cycles |
| --- | --- | --- |
| **Chair time** |  |  |
| Time per each cycle (hours) | 2,5 | 1 |
| Cost per each cycle (USD) | $165.7 | $95.6 |
| Cost per 1 patient (USD) | $497.2 | $1,433.4 |
| Cost per 1 patient, 18 cycles (USD) | $1,930.7 | |
|  |  |  |
| **Nurse time** |  |  |
| Time per each cycle (hours) | 2,5 | 1 |
| Cost per each cycle (USD) | $29.4 | $9.8 |
| Cost per 1 patient (USD) | $88.2 | $147 |
| Cost per 1 patient , 18 cycles (USD) | $235.3 | |
