# Supplementary material for: Cost-minimization analysis of subcutaneous versus intravenous trastuzumab administration in Chilean patients with HER2-positive early breast cancer
Source: PLoS One. 2020 Feb 5;15(2):e0227961. doi: 10.1371/journal.pone.0227961 (PMC7001963; doi:10.1371/journal.pone.0227961)
Supplement: S1 File — (ZIP) [file pone.0227961.s001.zip › S1 File/S6 Table.docx]

S6 Table. Cost of transportation, hourly salary and employment rate for the women´s workforce of 19 years and older in the metropolitan region of Chile

| Hourly salary (USD, 2017) | $ 4.7 |
| --- | --- |
| Round trip transportation cost (USD, 2017) | $ 2.1 |
| Employment rate (averange) | 62% |
